# Physics of Efficiency Droop in GaN:Eu Light-Emitting Diodes

**DOI:** 10.1038/s41598-017-17033-6

**Published:** 2017-12-01

**Authors:** Ioannis E. Fragkos, Volkmar Dierolf, Yasufumi Fujiwara, Nelson Tansu

**Affiliations:** 1 Center for Photonics and Nanoelectronics, Department of Electrical and Computer Engineering, Lehigh University, Bethlehem, PA 18015 USA; 2 Department of Physics, Lehigh University, Bethlehem, PA 18015 USA; 3 Division of Materials and Manufacturing Science, Graduate School of Engineering, Osaka University, Suita, Osaka, 565-0871 Japan

## Abstract

The internal quantum efficiency (IQE) of an electrically-driven GaN:Eu based device for red light emission is analyzed in the framework of a current injection efficiency model (CIE). The excitation path of the Eu^+3^ ion is decomposed in a multiple level system, which includes the carrier transport phenomena across the GaN/GaN:Eu/GaN active region of the device, and the interactions among traps, Eu^+3^ ions and the GaN host. The identification and analysis of the limiting factors of the IQE are accomplished through the CIE model. The CIE model provides a guidance for high IQE in the electrically-driven GaN:Eu based red light emitters.

## Introduction

In recent years, the incorporation of rare-earth (RE) elements in wide bandgap semiconductors, such as gallium nitride (GaN) has opened the way for light emitters in the wavelength range of the characteristic emission of the RE ion^1–5^. In particular, the incorporation of europium (Eu) into the GaN host (GaN:Eu) has attracted considerable attention because it enables red light emission and it has the potential to be used as an alternative to the low efficiency InGaN based red light emitters^6^. The excitation of the Eu^+3^ ion through the GaN host, results in light emission in the red spectral regime, and this has enabled the realization of GaN:Eu based devices in the last decade^7–24^. Despite the continues improvements of the GaN:Eu devices, the external quantum efficiency (η_EQE_) of the device exhibits a droop characteristic with increasing the current into the device^18,21–23,25^. The droop phenomenon of the external quantum efficiency (η_EQE_) needs to be clarified and suppressed in order to implement the GaN:Eu devices for technological applications. Therefore, the identification and understating of the limiting factors which result in the efficiency droop issue of the external quantum efficiency (η_EQE_) is crucial for the design and realization of high efficiency GaN:Eu based devices.

It is known that the excitation of the Eu^+3^ ions in the GaN host is mediated by traps which are close to the vicinity of the Eu^+3^ ions^8,9,21,26–29^. The injected electron-hole pairs in the GaN host are captured from these traps where they recombine and release energy. The released energy is used for the excitation of the nearby Eu^+3^ ion. The excitation path of the Eu^+3^ ion is a complex process since different carrier processes are involved, specifically including carrier transport across the GaN:Eu device, fundamental recombination processes of carriers in the GaN host, and interactions between the host, traps and Eu^+3^ ions. To the best of our knowledge, an analytical model which can describe the efficiency of the electrically-driven GaN:Eu device in the basis of the different processes and mechanisms involved in the Eu^+3^ excitation path, has not been developed until recently^30^.

In our recent work, we developed a current injection efficiency model (CIE) both for optically-pumped and electrically-driven GaN:Eu based device with an Al_x_Ga_1−x_N/GaN:Eu/Al_x_Ga_1−x_N quantum well (QW) active region^30^. In this work, we develop a CIE model for electrically-driven GaN:Eu device with a GaN/GaN:Eu/GaN active region to identify the limiting factors of the internal quantum efficiency (η_IQE_) and explain the efficiency droop issue of this type of active region. This type of structure is fundamentally different from the structure investigated in our previous work on quantum well based active region^30^. The present model provides the analysis of the current injection efficiency and IQE in the structures pursued by experimentalists^23^, specifically the active region with GaN/GaN:Eu where the active regions (GaN:Eu) were not confined by larger bandgap barrier systems. Such structure presented a very different challenge, which also required a completely different physics of carrier transport – beyond the QW model^30^. This present model is important for enabling the direct comparison with the experimental devices, and providing new strategies (see Section IV) for the experimentalists to increase the IQE and suppress the droop issue in electrically-driven rare-earth doped GaN LED.

## Current Injection Efficiency Model of the GaN:Eu active region

It is known that traps present in the vicinity of Eu^+3^ ions assist the excitation of the Eu^+3^ ion for red light emission. More specifically, studies have revealed several emission sites related to different configurations of trap-Eu^+3^ ion known as complexes^26,28^. In our model, we simplify this picture assuming a single level trap located near to the Eu^+3^ ion.

**Figure 1:**
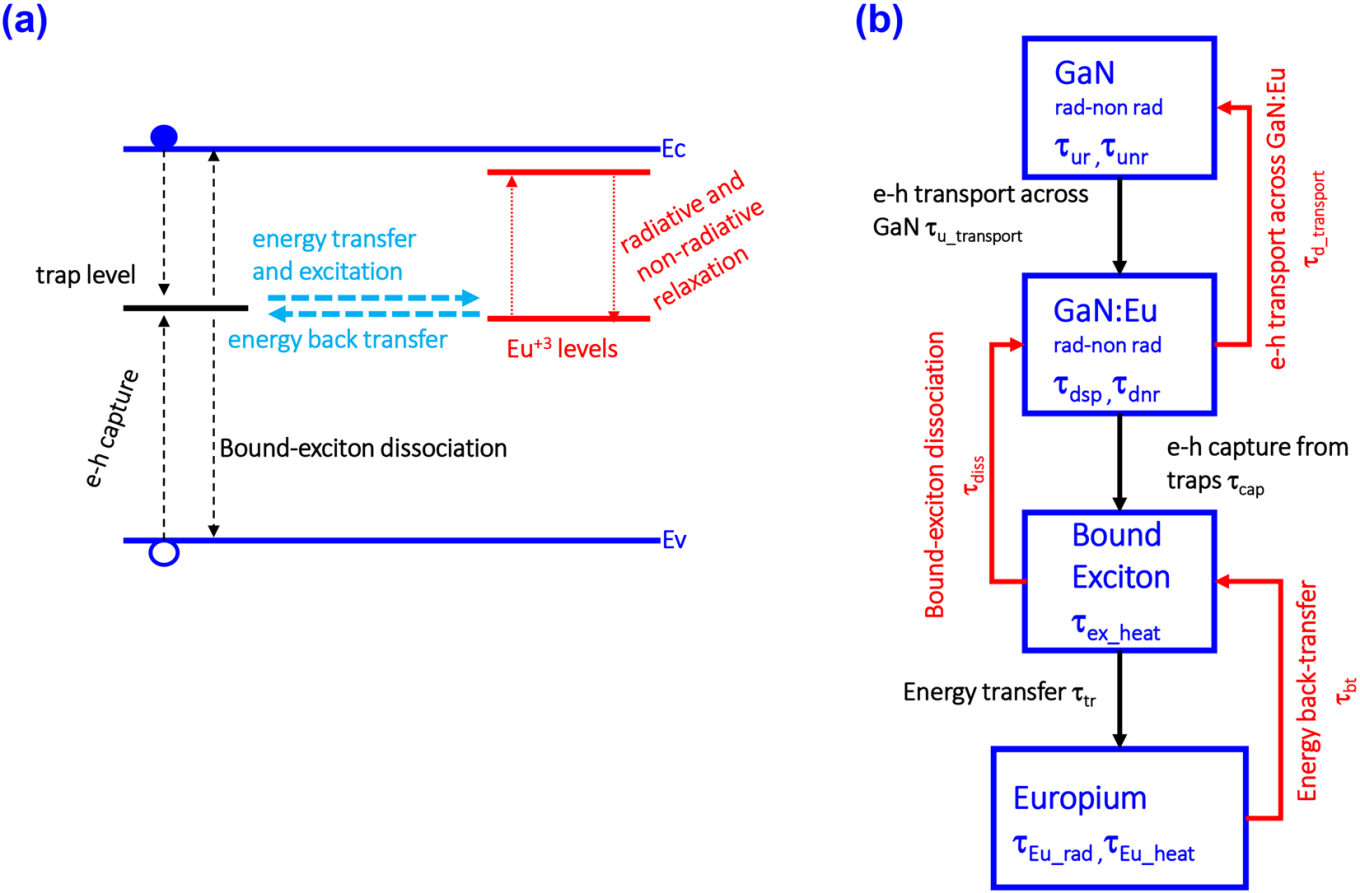
(**a**) Trap assisted excitation path of Eu^+3^ ion in the GaN host. The free electron-hole pairs present in the GaN host are captured by traps close to the vicinity of Eu^+3^ ions and form bound-excitons. The recombination of bound-excitons can result to energy transfer and excitation of a nearby Eu^+3^ ion. The excited Eu^+3^ ion can de-excite non-radiatively and radiatively as well as non-radiatively by releasing the energy to a nearby trap to form a bound-exciton. (**b**) Different levels and processes in the GaN/GaN:Eu/GaN active region structure. Each level includes its own related mechanisms, i.e radiative and non-radiative recombination processes for the “GaN” and “GaN:Eu” levels (Auger, Bimolecular and Shockley-Hall-Read recombination), de-excitation process with heat release to the crystal lattice for the “Bound-Exciton” level and radiative and non-radiative processes for the “Europium” level. The individual levels are connected through the “forward mechanisms” (black-arrows) and “backward mechanisms” (red-arrows).

As shown in Fig. 1(a), the free electron-hole pairs (e-h) present in the GaN host are captured by traps with a characteristic rate of 1/τ_cap_. This state of captured electron-hole pairs at the trap level is denoted as bound-exciton formation in our model. The recombination of e-h pair at the trap level can result in the energy transfer and excitation to a nearby Eu^+3^ ion (1/τ_tr_). In addition, different processes can take place at the trap level, including the non-radiative recombination of e-h pairs, which results in heat transfer to the lattice (1/τ_ex_heat_), as well as the bound-exciton dissociation process which leads to the release of the electron and hole back to the conduction band and valence band respectively (1/τ_diss_). The de-excitation process of the Eu^+3^ ion consists of the radiative (1/τ_rad_) and non-radiative (1/τ_Eu_heat_) processes, as well as the energy back-transfer process resulting in a formation of a bound-exciton (1/τ_bt_).

Figure 1(b) depicts the GaN/GaN:Eu/GaN active region structure and the related carrier mechanisms along the Eu^+3^ excitation path. According to our model, radiative and non-radiative recombination processes of carriers exist both in GaN and GaN:Eu regions. These fundamental processes are described through the Auger and the Shockley-Hall-Read (SHR) recombination processes (non-radiative) and the bimolecular recombination process (radiative) in the semiconductors. In addition, carrier transport mechanisms across the structure are present which are described through the ambipolar diffusion carrier transport across the GaN and GaN:Eu regions^31–34^.

The development of the current injection efficiency model for the GaN:Eu/GaN based LED is developed here, and the framework follows the treatments presented for III-V^31,32^ and GaN-based^33^ lasers/LEDs. The rate equations of carriers in the GaN region (N_1_) and GaN:Eu region (N_2_) are given by:

1 $$\frac{{{\rm{dN}}}_{1}}{{\rm{dt}}}=\frac{{{\rm{I}}}_{{\rm{tot}}}}{{{\rm{q}}{\rm{V}}}_{{\rm{1}}}}+\frac{{{\rm{N}}}_{2}}{{{\rm{\tau }}}_{{\rm{r}}2}}\frac{{{\rm{V}}}_{{\rm{2}}}}{{{\rm{V}}}_{{\rm{1}}}}-{{\rm{N}}}_{1}(\frac{1}{{{\rm{\tau }}}_{{\rm{nr}}1}}+\frac{1}{{{\rm{\tau }}}_{{\rm{sp}}1}}+\frac{1}{{{\rm{\tau }}}_{{\rm{r}}1}}),$$

2 $$\frac{{{\rm{dN}}}_{2}}{{\rm{dt}}}=\frac{{{\rm{N}}}_{1}}{{{\rm{\tau }}}_{{\rm{r}}1}}\frac{{{\rm{V}}}_{{\rm{1}}}}{{{\rm{V}}}_{{\rm{2}}}}+\frac{{{\rm{N}}}_{{\rm{ex}}}}{{{\rm{\tau }}}_{{\rm{diss}}}}-{{\rm{N}}}_{2}(\frac{1}{{{\rm{\tau }}}_{{\rm{nr}}2}}+\frac{1}{{{\rm{\tau }}}_{{\rm{sp}}2}}+\frac{1}{{{\rm{\tau }}}_{{\rm{r}}2}}+\frac{1}{{{\rm{\tau }}}_{{\rm{cap}}}}),$$

with the rate 1/τ_cap_ defined as

3 $$\frac{1}{{{\rm{\tau }}}_{{\rm{cap}}}}=\frac{1}{{{\rm{\tau }}}_{{\rm{cap}}0}}(1-\frac{{{\rm{N}}}_{{\rm{ex}}}}{{{\rm{N}}}_{{\rm{traps}}}}).$$

The I_tot_ is the total current injected into the GaN region from the n- and p-cladding layers of device. The parameters, N_ex_ and N_traps_, denote the bound-excitons and the maximum available trap concentration in the GaN:Eu region, respectively, and the τ_cap0_ is the capture time at the low regime where N_ex_≪N_traps_. In equations (1) and (2), the V_1_ and V_2_ are the volumes of the GaN and GaN:Eu regions respectively. The rates 1/τ_nr1_, 1/τ_sp1_, 1/τ_nr2_ and 1/τ_sp2_ are the non-radiative and spontaneous radiative recombination rates in the GaN (subscript 1) and GaN:Eu (subscript 2) regions, respectively. These rates are described by the SHR recombination constants A, the Auger coefficient C, and the bimolecular recombination constant B in the semiconductors. The rates 1/τ_r1_ and 1/τ_r2_ are described by the ambipolar diffusion carrier transport time in the GaN and GaN:Eu regions respectively.

The rate equations of bound-excitons and excited Eu^+3^ ion concentration are given by:

4 $$\frac{{{\rm{dN}}}_{{\rm{ex}}}}{{\rm{dt}}}={{\rm{N}}}_{2}\frac{1}{{{\rm{\tau }}}_{{\rm{cap}}}}+{{\rm{N}}}_{{\rm{Eu}}}\frac{1}{{{\rm{\tau }}}_{{\rm{bt}}}}-{{\rm{N}}}_{{\rm{ex}}}(\frac{1}{{{\rm{\tau }}}_{{\rm{tr}}}}+\frac{1}{{{\rm{\tau }}}_{{\rm{diss}}}}+\frac{1}{{{\rm{\tau }}}_{{\rm{ex}}\_{\rm{heat}}}}),$$

5 $$\frac{{{\rm{dN}}}_{{\rm{Eu}}}}{{\rm{dt}}}={{\rm{N}}}_{{\rm{ex}}}\frac{1}{{{\rm{\tau }}}_{{\rm{tr}}}}-{{\rm{N}}}_{{\rm{Eu}}}(\frac{1}{{{\rm{\tau }}}_{{\rm{bt}}}}+\frac{1}{{{\rm{\tau }}}_{{\rm{rad}}}}+\frac{1}{{{\rm{\tau }}}_{{\rm{Eu}}\_{\rm{heat}}}}),$$

The rates 1/τ_tr_ and 1/τ_bt_ are defined in an equivalent manner as in equation (3):

6 $$\frac{1}{{{\rm{\tau }}}_{{\rm{tr}}}}=\frac{1}{{{\rm{\tau }}}_{{\rm{tr}}0}}(1-\frac{{{\rm{N}}}_{{\rm{Eu}}}}{{\rm{N}}}),$$

7 $$\frac{1}{{{\rm{\tau }}}_{{\rm{bt}}}}=\frac{1}{{{\rm{\tau }}}_{{\rm{bt}}0}}(1-\frac{{{\rm{N}}}_{{\rm{ex}}}}{{{\rm{N}}}_{{\rm{traps}}}}),$$

where the N_Eu_ and N are the excited and the maximum concentrations of Eu^+3^ ions in the GaN:Eu region, respectively.

The current injection efficiency (η_injection_) is defined as the ratio of the current arising from the radiative and non-radiative de-excitation of Eu^+3^ ions over the total current I_tot_, entering the device:

8 $${{\rm{\eta }}}_{{\rm{injection}}}=\frac{{{\rm{I}}}_{{\rm{Eu}}}}{{{\rm{I}}}_{{\rm{tot}}}}.$$

The I_Eu_ corresponds to the total recombination current arising from the radiative and non-radiative de-excitation of the Eu^+3^ ion in the GaN:Eu region:

9 $${{\rm{I}}}_{{\rm{Eu}}}=\frac{{{\rm{N}}}_{{\rm{Eu}}}{{\rm{q}}{\rm{V}}}_{{\rm{Eu}}}}{{\rm{\tau }}},$$

with q as the electron charge, and the lifetime has both contributions from the radiative and non-radiative processes as stated below:

10 $$\frac{1}{{\rm{\tau }}}=\frac{1}{{{\rm{\tau }}}_{{\rm{rad}}}}+\frac{1}{{{\rm{\tau }}}_{{\rm{Eu}}\_{\rm{heat}}}},$$

By solving the rate equations (1), (2), (4), and (5) under steady state conditions and using the equation (8), the current injection efficiency for the GaN:Eu / GaN LED can be expressed as:

11 $${{\rm{\eta }}}_{{\rm{injection}}}={[(\frac{{{\rm{L}}}_{1}}{{{\rm{L}}}_{2}})(b+\frac{b}{{(\frac{1}{{{\rm{\tau }}}_{{\rm{r}}1}}+\frac{1}{{{\rm{\tau }}}_{{\rm{sp}}1}}+\frac{1}{{{\rm{\tau }}}_{{\rm{nr}}1}})}^{-1}}-(\frac{{{\rm{L}}}_{2}}{{{\rm{L}}}_{1}})\frac{c}{{{\rm{\tau }}}_{{\rm{r}}2}})]}^{-1}$$

with

12 $$b=\frac{{{\rm{L}}}_{2}}{{{\rm{L}}}_{1}}(a-\frac{{(\frac{1}{{{\rm{\tau }}}_{{\rm{rad}}}}+\frac{1}{{{\rm{\tau }}}_{{\rm{Eu}}\_{\rm{heat}}}})}^{-1}{{\rm{\tau }}}_{{\rm{tr}}}}{{(\frac{1}{{{\rm{\tau }}}_{{\rm{rad}}}}+\frac{1}{{{\rm{\tau }}}_{{\rm{Eu}}\_{\rm{heat}}}}+\frac{1}{{{\rm{\tau }}}_{{\rm{bt}}}})}^{-1}{{\rm{\tau }}}_{{\rm{diss}}}}){{\rm{\tau }}}_{{\rm{r}}1}$$

13 $$a=(\frac{c}{{(\frac{1}{{{\rm{\tau }}}_{{\rm{r}}2}}+\frac{1}{{{\rm{\tau }}}_{{\rm{sp}}2}}+\frac{1}{{{\rm{\tau }}}_{{\rm{nr}}2}}+\frac{1}{{{\rm{\tau }}}_{{\rm{cap}}}})}^{-1}})$$

14 $$c=((\tfrac{{(\tfrac{1}{{{\rm{\tau }}}_{{\rm{rad}}}}+\tfrac{1}{{{\rm{\tau }}}_{{\rm{Eu}}\_{\rm{heat}}}})}^{-1}{{\rm{\tau }}}_{{\rm{tr}}}}{{(\tfrac{1}{{{\rm{\tau }}}_{{\rm{rad}}}}+\tfrac{1}{{{\rm{\tau }}}_{{\rm{Eu}}\_{\rm{heat}}}}+\tfrac{1}{{{\rm{\tau }}}_{{\rm{bt}}}})}^{-1}{(\tfrac{1}{{{\rm{\tau }}}_{{\rm{diss}}}}+\tfrac{1}{{{\rm{\tau }}}_{{\rm{ex}}\_{\rm{heat}}}}+\tfrac{1}{{{\rm{\tau }}}_{{\rm{tr}}}})}^{-1}}-\tfrac{{(\tfrac{1}{{{\rm{\tau }}}_{{\rm{rad}}}}+\tfrac{1}{{{\rm{\tau }}}_{{\rm{Eu}}\_{\rm{heat}}}})}^{-1}}{{{\rm{\tau }}}_{{\rm{bt}}}}){{\rm{\tau }}}_{{\rm{cap}}}),$$

where, the L_1_ and L_2_ are the lengths of the GaN and GaN:Eu regions respectively. Then, the internal quantum efficiency (η_IQE_) of the rare-earth doped GaN LED is given by:

15 $${{\rm{\eta }}}_{{\rm{IQE}}}={{\rm{\eta }}}_{\text{injection}}\cdot {{\rm{\eta }}}_{{\rm{rad}}},$$

where, the η_rad_ is the radiative efficiency of the Eu^+3^ ions defined as the ratio of radiative to both radiative and non-radiative de-excitation of Eu^+3^ ions:

16 $${{\rm{\eta }}}_{{\rm{rad}}}=\frac{{{\rm{N}}}_{{\rm{Eu}}}/{{\rm{\tau }}}_{{\rm{rad}}}}{{{\rm{N}}}_{{\rm{Eu}}}/{\rm{\tau }}}=\frac{\frac{1}{{{\rm{\tau }}}_{{\rm{rad}}}}}{\frac{1}{{{\rm{\tau }}}_{{\rm{rad}}}}+\frac{1}{{{\rm{\tau }}}_{{\rm{Eu}}\_{\rm{heat}}}}}.$$

## Simulation Results

Table 1 summarizes the parameters used for each numerical calculation. The material parameters used for the simulations, such as SHR recombination constant A, bimolecular recombination constant B, Auger coefficient C, electron and hole effective mases and mobilities can be found in ref.^33^. For this work we assume the same values of material parameters both for the GaN and GaN:Eu region. In addition, for the magnitude of the relative times related to traps, bound-excitons and Eu^+3^ ions, the experimental results described in^35,36^ were used as a point of reference.

**Table 1:** Parameters used for the numerical calculations.

Parameters	Study I	Study II	Study III	Study V	Study VI	Study VII
A (10^8^ s^−1^)	0.001–1	0.1	0.1	0.1	0.1	0.1
τ_cap0_ (10^−8^ s)	100	100	1-1000	100	100	100
τ_tr0_ (10^−7^ s)	360	360	360	0.36–3600	360	360
τ_diss_ (10^−6^ s)	1000	1000	1000	1000	1000	1000
τ_bt0_ (10^−6^ s)	200	200	200	200	200	200
τ_ex_heat_ (10^–3^ s)	1	1	1	1	1	1
τ_Eu_heat_ (10^−3^ s)	1	1	1	1	1	1
τ_rad_ (10^−6^ s)	400	400	400	400	10–600	400
N (10^19^ cm^−3^)	1	1	1	1	0.1–10	0.1–10
N_traps_ (10^19^ cm^−3^)	1	1	1	1	0.1–10	0.1–10
L_GaN_, L_GaN:Eu_ (nm)	5, 2.5	2.5–6.5, 2.5–6.5	5, 2.5	5, 2.5	5, 2.5	QW region

For each study, the current injection efficiency (η_injection_) and the excited Eu^+3^ ion concentration are plotted versus the current density J entering the GaN:Eu device. From our studies, the current injection efficiencies (η_injection_) of the GaN:Eu LEDs exhibit the drooping characteristics with increasing current density J. This droop characteristic, which is present in the case studies, arises from the saturation of the excited Eu^+3^ ions in the active region. The excited Eu^+3^ ion concentration cannot exceed the maximum available concentration (N) in the GaN:Eu region. Therefore, further increasing the injected current density into the device result in a bottleneck in the energy transfer from bound-excitons into the Eu^+3^ ions, which decreases the current injection efficiency (η_injection_). It is important to note that the rate of this saturation and the subsequent droop in the current injection efficiency (η_injection_) are strongly dependent on the different parameters of the Eu^+3^ excitation path. It is essential to point out that the effect of Auger recombination in our studies is expected to be negligible due to the relatively low carrier concentration of ~10^18^ cm^−3^ in the active region for electrically-driven GaN:Eu LEDs (J ~ 10 A/cm^2^).

### Effect of Shockley-Hall-Read constant A

**Figure 2:**
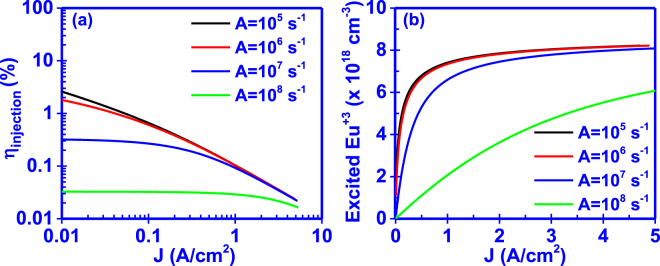
Effect of Shockley-Hall-Read constant A on the (**a**) current injection efficiency (η_injection_) and (**b**) excited Eu^+3^ ion concentration of the GaN:Eu active region.

The Shockley-Hall-Read constant A (SHR), is related to defects which are present in the crystal lattice and are not related to the incorporation of the Eu^+3^ ions in the GaN host, thus do not assist the Eu^+3^ excitation. However, they capture free carriers and contribute to the non-radiative recombination process. As shown in Fig. 2a and b, decreasing the SHR constant A results in higher current injection efficiencies (η_injection_) as well as in faster saturation rates of the excited Eu^+3^ ion concentration. The low values of A indicate lower non-radiative recombination rates of carriers, thus more carriers are available to contribute to the excitation of Eu^+3^ ions. Furthermore, decreasing the A below A=10^6^ s^−1^, results in a minimal effect on the current injection efficiency (η_injection_) and exited Eu^+3^ ion concentration for the given range of input current densities J of the device.

### Effect of GaN and GaN:Eu region lengths

**Figure 3:**
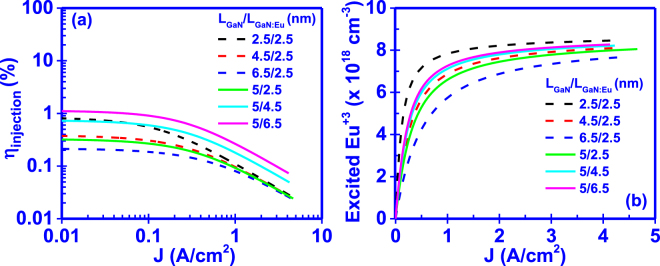
Effect of GaN and GaN:Eu region lengths (L_GaN_/L_GaN:Eu_) on (**a**) current injection efficiency (η_injection_) and (**b**) excited Eu^+3^ ion concentration of the GaN:Eu region. The dashed lines correspond to changes in the length of the GaN region (L_GaN_) with a fixed L_GaN:Eu_ = 2.5 nm. Similarly, the solid lines corresponds to changes in the length of the GaN:Eu region (L_GaN:Eu_) with a fixed L_GaN_ = 5 nm.

The effect of the lengths of the GaN and GaN:Eu regions is depicted in Fig. 3. As it is shown, the increase of the GaN length (L_GaN_) reduces the current injection efficiency (η_injection_) and excited Eu^+3^ ion concentration. In contrast, an increase in the GaN:Eu length (L_GaN:Eu_) will give rise to the current injection efficiency (η_injection_) as well as to the excited Eu^+3^ ion concentration under steady state conditions. The change in the lengths of the two regions affects the ambipolar diffusion transport time (τ_r1_, τ_r2_) of carries across the structure. For higher L_GaN_, the carriers require more time to be transported across the GaN region, reducing the rate at which they arrive in the GaN:Eu region. As a result, the current injection efficiency (η_injection_) and excited Eu^+3^ ion concentration in the active region are decreased. In contrast, for higher L_GaN:Eu_, the ambipolar diffusion transport time in the GaN:Eu region (τ_r2_) will be increased giving rise to the carrier concentration in the GaN:Eu region. The increased carrier concentration in the GaN:Eu region will result in higher probability of bound-exciton formation. Consequently, the excited Eu^+3^ ion concentration and the current injection efficiency (η_injection_) are increased.

### Effect of capture time

**Figure 4:**
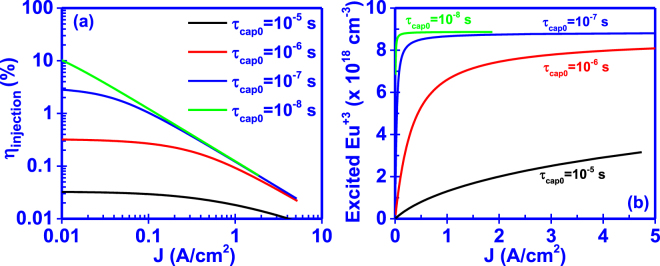
Effect of capture time (τ_cap0_) on the (**a**) current injection efficiency (η_injection_) and (**b**) excited Eu^+3^ ion concentration of the GaN:Eu active region.

The capture time from traps (τ_cap0_), which are associated with the incorporation of Eu^+3^ ions, defines the rate at which bound-excitons are formed by capturing e-h pairs from the GaΝ host. It is clearly seen from Fig. 4a and b that decreasing the capture time, the current injection efficiency (η_injection_) increases while the Eu^+3^ ion concentration saturates faster. From equation (4), the lower capture time results in a higher formation rate of bound-excitons which in turn gives rise to the excitation rate of Eu^+3^ ions under steady state conditions.

In addition, for the cases with capture times lower than τ_cap0_ = 10^−7^ s, the changes in the current injection efficiency (η_injection_) are prominent at low current densities, which can be attributed to the faster saturation of the excited Eu^+3^ ion concentration. Despite the faster capture rate τ_cap0_ of carriers form traps, the saturation of the excited Eu^+3^ ion will remain the bottleneck in the injection process in high current density regime.

### Effect of transfer time

**Figure 5:**
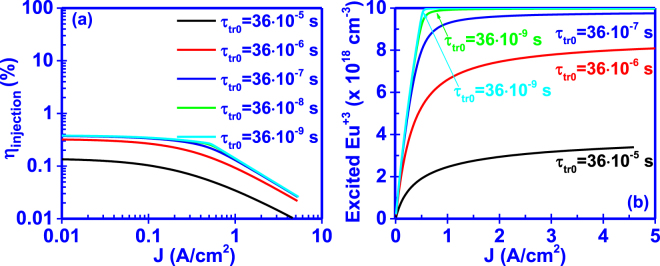
Effect of transfer time (τ_tr0_) on the (**a**) current injection efficiency (η_injection_) and (**b**) excited Eu^+3^ ion concentration of the GaN:Eu active region.

The excitation process of the Eu^+3^ ions is driven by the energy transfer from the bound-excitons formed by the captured e-h pairs in the traps. This transfer rate (1/τ_tr0_) is dictated by the strength of the interaction between the bound-excitons and Eu^+3^ ions. Figure 5a and b depict the effect of the transfer time in the current injection efficiency (η_injection_) and excited Eu^+3^ ion concentration, respectively. As the transfer time decreases, the current injection efficiency (η_injection_) increases accompanied by the higher saturation of the excited Eu^+3^ ion up to higher current density level. However, a decrease of transfer time bellow τ_tr0_ = 36 × 10^–7^ s does not have a significant effect on the current injection efficiency (η_injection_) since the excited Eu^+3^ ion concentration remains almost unaffected under further reduction of the transfer time (τ_tr0_).

### Effect of radiative lifetime of Eu^+3^ ion

The changes in the trap capture, transport, transfer, and recombination rates – as discussed above–show the ability to engineer the current injection efficiency in RE-doped GaN LEDs at the low injection current density level. The optimized structure – based on the results above - enables up to η_injection_ ~10% at low injection current density level, while our finding shows that the engineering of the radiative lifetime for the RE-ions will be instrumental in governing the current injection efficiency properties at high current density level.

To alter the current injection efficiency (η_injection_) at higher input current densities it is necessary to delay the saturation of Eu^+3^ ions. This can be accomplished by reducing the radiative lifetime of Eu^+3^ ions (τ_rad_). From equations (9) and (10) the lower radiative lifetime (τ_rad_) of Eu^+3^ ions will increase the radiative recombination rate and also the current injection efficiency (η_injection_) at a particular input current. Furthermore, the radiative efficiency (η_rad_) of the system will be increased, giving additional rise to the internal quantum efficiency (η_IQE_) of the active region.

**Figure 6:**
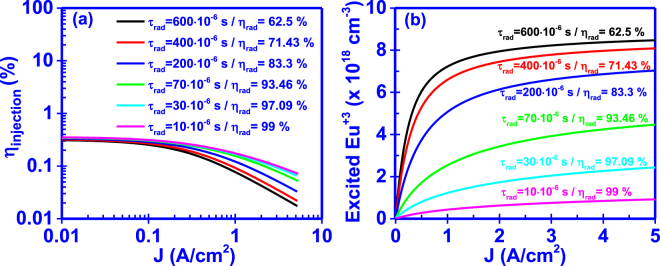
Effect of radiative lifetime of Eu^+3^ ion (τ_trad_) on the (**a**) current injection efficiency (η_injection_) and (**b**) excited Eu^+3^ ion concentration of the GaN:Eu active region. Each radiative lifetime results in different radiative efficiency of the excited Eu^+3^ ions.

As shown in Fig. 6a and b, reducing the radiative lifetime (τ_rad_) of Eu^+3^ ions results in the increased current injection efficiency (η_injection_) at higher input current densities. This arises from the lower saturation values of excited Eu^+3^ ions at the given input current density range, as shown in Fig. 6(b). A change of the radiative lifetime from τ_rad_ = 600 μs to τ_rad_ = 10 μs, at a current density of J = 4 A/cm^2^, will result in an increase of 214% in the current injection efficiency (η_injection_) and in a reduction of 90% in the excited of Eu^+3^ ion concentration in the active region.

### Effect of carrier confinement in the GaN:Eu region

**Figure 7:**
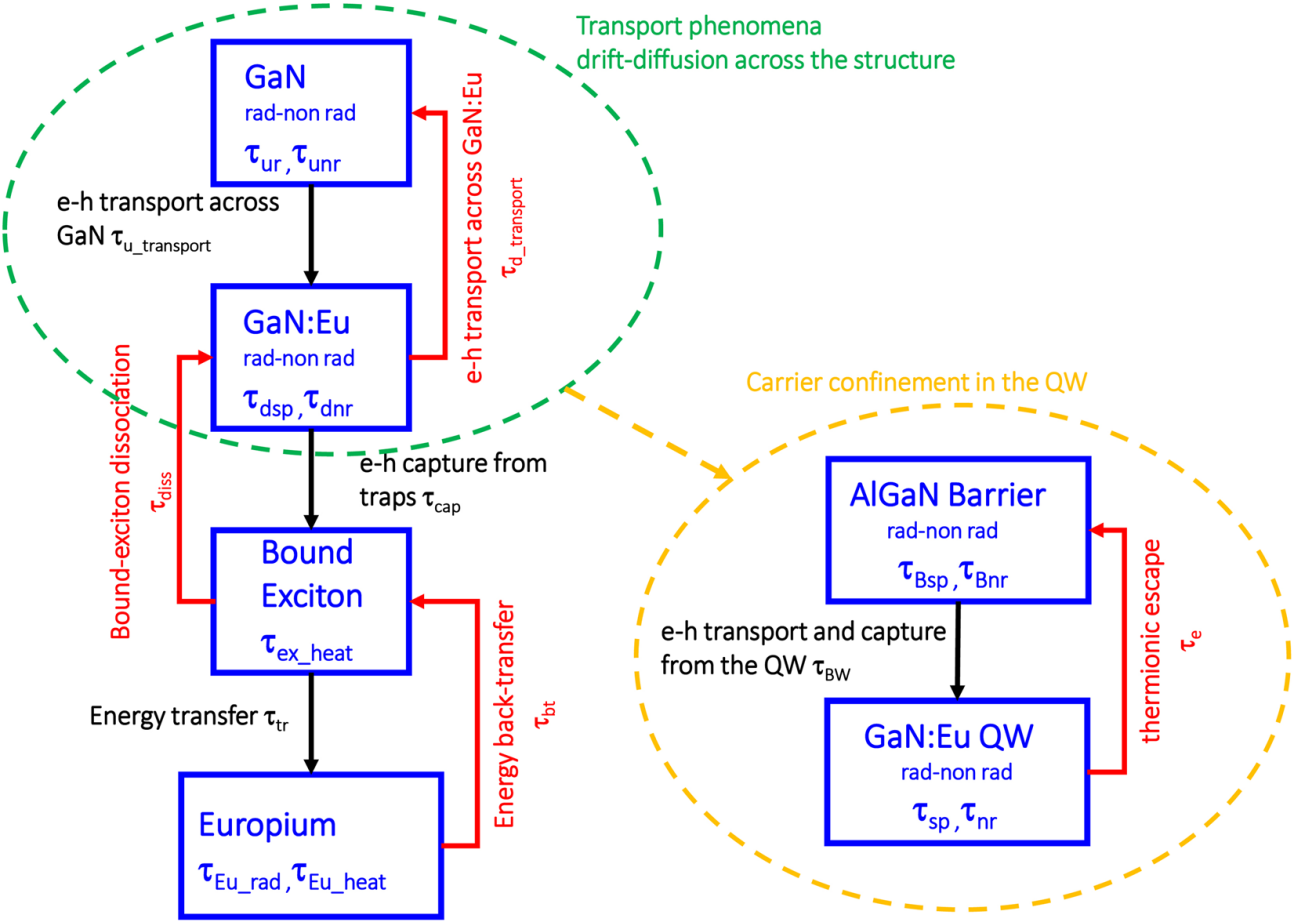
Active region structures for a GaN:eu based device. The effect of the Al_x_Ga_1−x_N/GaN:Eu/Al_x_Ga_1−x_N heterostructure results in the formation of a GaN:Eu quantum well (QW). This QW structure results in quantum mechanical processes such as the capture of carriers form the barrier to the QW as well as to the thermionic carrier escape form the QW to the barrier. The carrier confinement give rise to the carrier concentration inside the GaN:Eu QW which in turns enhances the excitation probability of Eu^+3^ ions.

The utilization of heterostructures such as Al_x_Ga_1−x_N/GaN:Eu/Al_x_Ga_1−x_N increases the carrier confinement in the GaN:Eu quantum well (QW) region^30^, enhancing in that way the excitation probability of Eu^+3^ ions. In addition, the replacement of the GaN/GaN:Eu/GaN structure with the Al_x_Ga_1−x_N/GaN:Eu/Al_x_Ga_1−x_N heterostructure results in different carrier processes (Fig. 7). These additional processes include the quantum mechanical capture process of carriers from the barrier to the QW and the thermionic carrier escape process from the GaN:Eu QW to the Al_x_Ga_1−x_N barriers.

**Figure 8:**
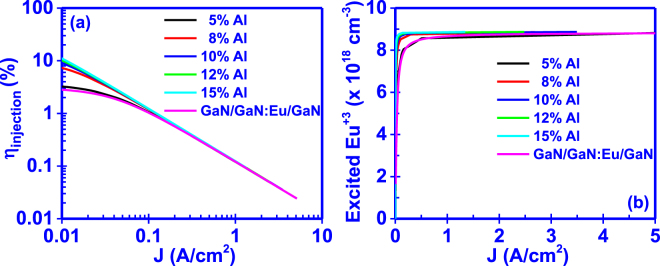
Effect of carrier confinement on (**a**) current injection efficiency (η_injection_) and (**b**) excited Eu^+3^ ion concentration of the GaN:Eu active region. Higher Al percentage in the Al_x_Ga_1−x_N barrier results in the supression of the thermionic emission of carriers form the GaN:Eu QW to the Al_x_Ga_1−x_N barrier.

The effect of the carrier confinement on the current injection process in RE-doped GaN LED is shown in Fig. 8. The presence of the GaN:Eu quantum well (QW) confined within Al_x_Ga_1−x_N barriers increases the current injection efficiency (η_injection_) and the excited Eu^+3^ ion concentration at a given current density. The carrier confinement in the QW increases the carrier density near the Eu^+3^ ions and thus, the probability of carrier capture form traps increases, giving rise to the excitation of Eu^+3^ ions. In addition, increasing the Al composition of the Al_x_Ga_1−x_N barrier increases the barrier height, which results in the suppression of the thermionic carrier escape process^33^. As a result, the current injection efficiency (η_injection_) and excited Eu^+3^ ion concentration in the active region are increased. The effect of carrier confinement, has been demonstrated by T. Arai *et al*.^21^, where they showed an increase of the PL intensity of a AlGaN/GaN:Eu/AlGaN multiple QW structure as compared to a rudimentary GaN:Eu based light emitter. Similar findings have been demonstrated for Erbium-doped GaN based heterostructures, where the effect of carrier confinement increases the luminescence of the GaN:Er emitter^37,38^.

## Droop Suppressions

**Figure 9:**
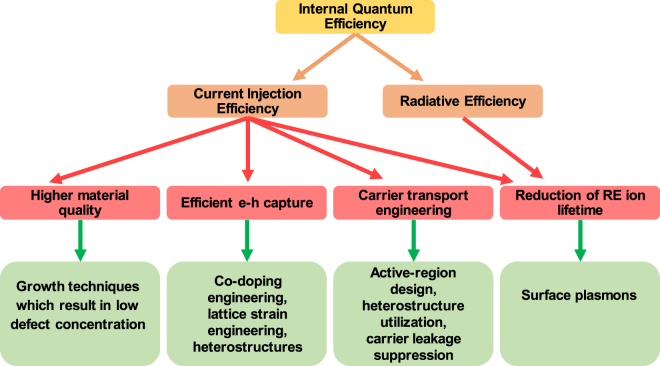
Internal Quantum Efficiency in the GaN:Eu based devices and its dependency on the parameters along the excitation path of Eu^+3^ ion.

Based on our analysis, we present several experimental pathways on how to increase the internal quantum efficiency (η_IQE_) of the GaN:Eu based devices, including strategies for suppressing droop. In Fig. 9, the internal quantum efficiency (η_IQE_) is decomposed into two components, namely the current injection (η_injection_) and radiative (η_rad_) efficiencies. The individual efficiencies depend on specific phenomena along the excitation path of Eu^+3^ ions. The experimental pathway on how to alter these phenomena, in favor of the respective efficiency, are also shown in Fig. 9.

The utilization of advanced growth techniques such as metalorganic chemical vapor deposition technique (MOCVD) can result in high crystal quality^39–42^. By carefully adjusting the growth parameters, the defect concentration in the GaN host can be minimized resulting in lower SHR recombination constant A. In addition, the lower defect concentration will give rise to the carrier mobility along the structure due to reduction of the scattering centers. The carrier mobility affects the transport time, which plays an essential role in the electrically-driven internal quantum efficiency (η_IQE_) of the system. The direct effect of carrier mobility was not presented here, but it is evident through the ambipolar diffusion transport time^31–34^. For higher carrier mobility in the GaN region, the current injection efficiency (η_injection_) will be enhanced in the RE-doped LEDs. In contrast, the higher carrier mobility in the GaN:Eu region will result in lower current injection efficiency (η_injection_) and lower excited Eu^+3^ ion concentration in the active region. Both the carrier mobility and length of the device active regions affect the transport time. Furthermore, the utilization of heterostructure will be beneficial for the internal quantum efficiency (η_IQE_), attributed to the stronger carrier localization which in turn increases the trap capture probabilities^21,37,38^. In order to obtain a more efficient excitation of Eu^+3^ ion, co-doping and strain engineering in the GaN host are possible pathways. These methods have been proved to result in more efficient capture process and energy transfer process to the Eu^+3^ ion^18,23,25,26,28,29,43^.

The effect of radiative lifetime of Eu^+3^ ions is crucial for the internal quantum efficiency (η_IQE_) of the device. From our analysis, the current injection efficiency (η_injection_) and consequently the internal quantum efficiency (η_IQE_) are limited by the saturation of the excited Eu^+3^ ion concentration at higher input current densities. In order to achieve higher efficiencies at higher input current densities, the change of this saturation rate is essential. It has been experimentally demonstrated that by utilizing surface-plasmon (SP) in GaN-based QW can significantly increase the radiative efficiency of the system^44–47^. For the case of GaN:Eu based emitters, through the engineering of the deposited materials used as SPs, the SP frequency can be adjusted to be very closed to the frequency of the emitted photons from the Eu^+3^ ions. This approach will increase both the current injection efficiency (η_injection_) and also the radiative efficiency (η_rad_) of the system.

## Comparison with experiment

The results from our CIE model are compared with the experimentally reported values. More specifically, we calculated the external quantum efficiency (η_EQE_) of a GaN/GaN:Eu/GaN structure. The external quantum efficiency (η_EQE_) is defined as the product of the internal quantum efficiency (η_IQE_) and the extraction efficiency (η_EXT_) of the device. For the purpose of these calculations, a device area of 0.1 × 0.1 cm^2^ with an external quantum efficiency of η_EXT_ = 44% was used, which is a typical value of the GaN:Eu based devices^22^. W. Zhu and co-workers fabricated a high power GaN:Eu based LED via low temperature MOCVD technique^23^. The active region of this device consisted of alternate GaN (6 nm) and GaN:Eu (3 nm) regions and exhibited an external quantum efficiency of η_EQE_ = 4.6% at an injected current of 1 mA which was reduced to η_EQE_ = 0.9% at 20 mA. These values correspond to the highest reported external quantum efficiencies (η_EQE_) for a GaN:Eu based device up to date.

**Figure 10:**
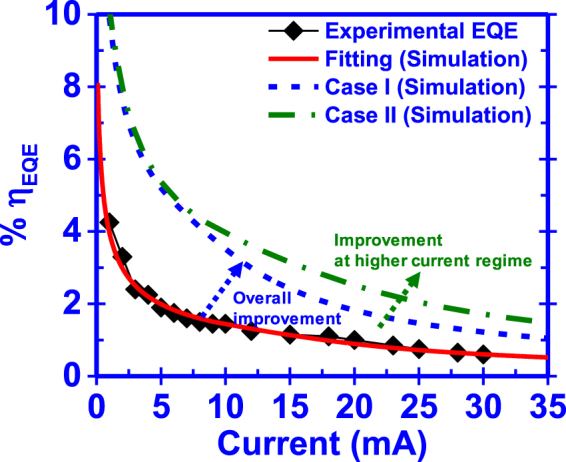
Experimentally reported values for a GaN:Eu device and the fitting with the CIE model. Two simulations for higher external quantum efficiency are performed. The corresponding percentage increase at the particular current, is calculated with respect to the fitting of the experimental values.

Figure 10 presents our numerical fitting results to the experimentally reported values from W. Zhu *et al*.^23^. The simulation parameters used here are presented in Table 2. Our simulated results provided an excellent fit with the experimental data from ref.^23^, as shown in Fig. 10.

**Table 2:** Parameters used for the fitting of the experimental values of the GaN:Eu device and the simulations for higher external quantum efficiency (η_EQE_).

Parameters	Fitting	Case I	Case II
A (10^8^ s^−1^)	0.01	0.01	0.01
τ_cap0_ (10^−8^ s)	10	10	10
τ_tr0_ (10^−7^ s)	0.36	0.36	0.36
τ_diss_ (10^−6^ s)	1000	1000	1000
τ_bt0_ (10^−6^ s)	200	200	200
τ_c_heat_ (10^−3^ s)	1	1	1
τ_Eu_heat_ (10^−3^ s)	1	1	1
τ_rad_ (10^−6^ s)	100	100	70
N (10^19^ cm^−3^)	8.7	8.7	8.7
N_traps_ (10^19^ cm^−3^)	8.7	8.7	8.7
L_GaN_, L_GaN:Eu_ (nm)	6, 3	6,6	6,6

In order to guide the experiments, we investigated two cases (Case I and Case II) with different design parameters. In Case I (Fig. 10), by increasing the length of the GaN:Eu region from 3 nm to 6 nm, an increase of the external quantum efficiency (η_EQE_) with respect to the fitting of the experimental data is possible. More specifically, an increase of 167%, 112% and 103% at an injected current of 5 mA, 15 mA and 30 mA respectively, is predicted. In Case II, by an additional decrease of the radiative lifetime of Eu^+3^ ion from τ_rad_ = 100 μs to τ_rad_ = 70 μs, an increase of 173% and 183% at 15 mA and 30 mA respectively, is possible.

The experimental work by W. Zhu *et al*., showed that increasing the current into the GaN:Eu device, will eventually result in the saturation of the output light power of the device, as well as, in the decrease of the external quantum efficiency (η_EQE_). The saturation in the output power is a result of the saturation of the excited Eu^+3^ ions in the active region. Our work has showed this saturation causes the efficiency droop issue in the GaN:Eu devices. Similar results have been experimentally verified elsewhere^18,19,21^.

## Conclusion

In summary, we developed a current injection efficiency model (CIE) for a GaN:Eu based device with a GaN/GaN:Eu/GaN structure as an active region, in order to identify the limiting factors of the internal quantum efficiency (η_IQE_) of the GaN:Eu based device. The analysis of the internal quantum efficiency (η_IQE_) is accomplished in the basis of a multilevel system, which includes the carrier behavior and mechanisms in the GaN and GaN:Eu regions and the interactions of the traps, carriers and Eu^+3^ ions with the host. It was found that the droop in the efficiency of the GaN:Eu device is associated with the droop in the current injection efficiency (η_injection_) of the active region which arises from the saturation of the excited Eu^+3^ ion concentration in the active region. Through the manipulation of the characteristic rates and processes associated with the excitation path of Eu^+3^ ions, efficiencies higher than the current state of the art can be achieved. Our work demonstrates the pathway for enhancing the efficiency of the GaN:Eu based red light emitting devices. The CIE model can be extended to other RE-doped wide bandgap semiconductors, in which the excitation of RE ion is trap assisted.
